# Global Gene Expression Profiling of Individual Human Oocytes and Embryos Demonstrates Heterogeneity in Early Development

**DOI:** 10.1371/journal.pone.0064192

**Published:** 2013-05-22

**Authors:** Lisa Shaw, Sharon F. Sneddon, Leo Zeef, Susan J. Kimber, Daniel R. Brison

**Affiliations:** 1 Faculty of Medical and Human Sciences, University of Manchester, Manchester, United Kingdom; 2 Department of Reproductive Medicine, Old St Mary’s Hospital, Central Manchester University Hospitals NHS Foundation Trust, Manchester Academic Health Sciences Centre, Manchester, United Kingdom; 3 Faculty of Life Sciences, University of Manchester, Manchester, United Kingdom; Baylor College of Medicine, United States of America

## Abstract

Early development in humans is characterised by low and variable embryonic viability, reflected in low fecundity and high rates of miscarriage, relative to other mammals. Data from assisted reproduction programmes provides additional evidence that this is largely mediated at the level of embryonic competence and is highly heterogeneous among embryos. Understanding the basis of this heterogeneity has important implications in a number of areas including: the regulation of early human development, disorders of pregnancy, assisted reproduction programmes, the long term health of children which may be programmed in early development, and the molecular basis of pluripotency in human stem cell populations. We have therefore investigated global gene expression profiles using polyAPCR amplification and microarray technology applied to individual human oocytes and 4-cell and blastocyst stage embryos. In order to explore the basis of any variability in detail, each developmental stage is replicated in triplicate. Our data show that although transcript profiles are highly stage-specific, within each stage they are relatively variable. We describe expression of a number of gene families and pathways including apoptosis, cell cycle and amino acid metabolism, which are variably expressed and may be reflective of embryonic developmental competence. Overall, our data suggest that heterogeneity in human embryo developmental competence is reflected in global transcript profiles, and that the vast majority of existing human embryo gene expression data based on pooled oocytes and embryos need to be reinterpreted.

## Introduction

Development of the human embryo begins at fertilisation with fusion and reprogramming of the gamete pronuclei, followed by a series of cleavage stages and activation of the embryonic genome [1], [2], [3]. After embryonic genome activation (EGA) and cleavage compaction occurs, the blastocyst forms giving rise to the first differentiated tissues, the trophectoderm and inner cell mass [4]. Although early human development shares many features with other species, there are also some notable differences particularly in the timing of embryonic genome activation (EGA) which has been shown to occur at the two-cell stage in the mouse, four-cell stage in the pig and eight-to sixteen- cell stage in the sheep, cow and rabbit (for a comprehensive review see Telford et al [5]. In the human, EGA was thought to occur at the four-cell stage [1], [4], [6], however, Vassena et al, [3] has shown EGA may occur in the human embryo as early as the two-cell stage. Our current understanding of this phase of development is limited, and little is known about the molecular mechanisms that control the developmental programme which occurs following fertilisation.

This lack of knowledge is a major concern as there is increasing evidence that the genetic and epigenetic blueprint for development is laid down at the preimplantation stage. In this period, parental genomes are reconfigured and the new embryonic genome is activated, methylation imprints are re-established, and the earliest stages of foetal development occur. Data from animal models and human assisted reproduction technologies (ART) has established beyond doubt that this sensitive period is highly vulnerable to perturbation [7]. In ART, the lack of basic understanding of regulators of human embryo viability and health is hampering efforts to select and transfer a single embryo, reducing success rates, increasing risk to offspring, and continuing to expose women to the increased risk of multiple pregnancy from multiple embryo transfer. The inability to characterise normal human embryonic development also has implications for the safety and efficacy of human embryonic stem cell (hESC) technologies, especially with the recent development of these towards clinical therapies. Major aspects of health and disease in adult life are now also widely recognised to originate as early as the preimplantation embryonic stages, including diseases arising from aberrations in fetal programming [8] and aberrant genomic imprinting [9]–[12].

It is therefore essential that we begin to unravel the molecular basis of early human development. Studies to date have been hindered by the small size of the mammalian preimplantation embryo and in the case of the human, the lack of embryos available for research for ethical reasons. Global transcript profiling using microarrays has been widely used to provide insight into animal oocytes and their transition into early embryos [13]–[15] and this approach has also been applied to human embryos [16]–[22]. These studies have provided valuable baseline data, but have analysed pooled oocytes and embryos. Very recently, technological advances have allowed microarray technology to be applied to individual oocytes and embryos. Vassena et al. [3] have used single human oocyte and embryo samples to demonstrate that embryonic genome activation (EGA) is initiated in waves of transcriptional activation in early preimplantation development.

However, the defining feature of early human development is heterogeneity. In contrast to animal models, human embryos vary considerably in their developmental competence, as established by data from normal reproduction (e.g. miscarriage rates and pregnancy complications) and human ART programmes. This heterogeneity is also reflected in the limited number of molecular studies available to date on individual embryos at a range of stages of development [23]–[25]. Therefore any attempts to understand the molecular basis of human development need to analyse heterogeneity at the single embryo level.

The aim of this study was therefore to investigate the global gene expression profile at three key developmental stages spanning human preimplantation embryo development; the metaphase II oocyte; followed by the 4-cell stage at which point EGA has occurred in human preimplantation development, and then the blastocyst, the stage immediately prior to implantation. We have employed polyAPCR amplification and microarray technology to investigate the wide ranging expression of genes during preimplantation development at the single embryo level. Our data suggest that the heterogeneity in human embryo development is reflected in global transcriptional profiles, and that existing human embryo microarray data based on pooled oocytes and embryos need to be reinterpreted.

## Results

We have utilised and further developed PolyAPCR amplification technology to generate sufficient cDNA from individual human oocytes and preimplantation embryos [26 27] to allow global transcriptome profiling using cDNA microarrays for the key developmental stages spanning preimplantation development. The PolyAcDNA was generated from single human oocytes and embryos surplus to IVF requirements and hybridised to Affymetrix U133 microarray gene chips to assess the expression of 47 000 transcripts [28]. The microarray data were validated by additionally using quantitative-PCR to confirm expression of a number of key genes. *TP53, GAS5, POU5F1, NANOG, ZFP42, CDX2* and *EIF1AX* were called present in all blastocyst samples and exhibited variable expression in four-cell embryos. Expression of these genes was confirmed by qPCR (see [27] for further detail). Oocytes also showed variable expression of these genes which was also confirmed by qPCR (unpublished data).

We chose to analyse oocytes, 4-cell embryos and blastocysts in order to gauge the extent of expression of maternal transcripts, their degradation by the 4-cell stage, and expression of new transcripts following EGA. We analysed 3 oocytes/embryos from different donors, in order to achieve the goals of examining heterogeneity at a level of detail not possible with large sample sets. For ethical reasons the oocytes were only available 24 hours after insemination, hence their gene expression patterns could be slightly different to fresh oocytes obtained on the day of egg collection. However we have shown previously that the two sources of oocytes yield comparable embryos and human embryonic stem cell lines with similar gene expression patterns [29], [30].

**Figure 1:**
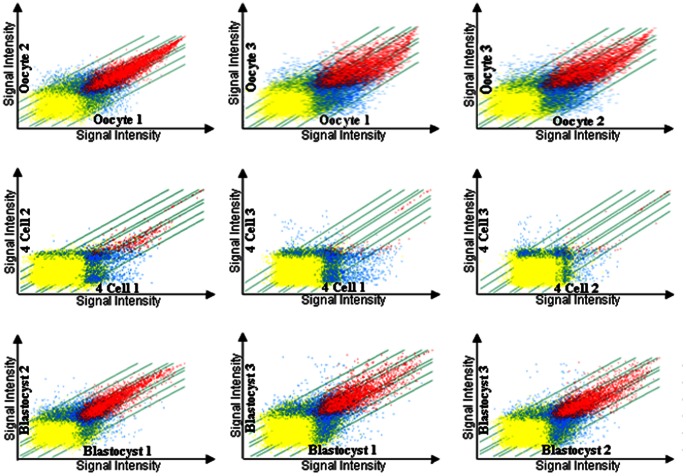
Scattergraph plots comparing gene expression of individual human oocytes, 4-cell embryos and blastocysts. Each of three individual samples at each stage is compared to each of the others. Red dots represent transcripts called Present in both samples, yellow dots represent transcripts absent in both samples and blue dots represent transcripts Present in one sample and not the other. The innermost oblique lines represent 2-fold differentially expressed transcripts. Additional pairs of lines represent transcripts expressed at 5, 10- and 20-fold, respectively. As expected, fewer genes were called Present at the 4-cell stage which reflects the degradation of polyA containing maternal transcripts by the 4-cell stage during EGA.

Comparison between individual human oocytes, 4-cell embryos and blastocysts shows that within each stage, global gene expression is broadly reproducible, as indicated by transcripts expressed in common between two samples at the same stage, compared to those expressed uniquely (Figure 1). A large number of transcripts were expressed in oocytes and blastocysts, with very few expressed at the 4-cell stage.

Our data also reveal significant variation amongst individual oocytes and embryos. Figure 1 shows increased variation in transcript expression in oocyte #3, 4-cell embryo #1 and blastocyst #3. Oocyte 1 and 2 expressed similar transcription profiles, with oocyte 3 expressing fewer unique transcripts than the others (Figure 1). Four-cell embryos 2 and 3 displayed less scatter when compared to each other, but each sample expressed largely unique transcripts with very few expressed in common (Figure 1). Blastocysts 1 and 2 showed a similar expression profile but blastocyst 3 showed a more differential expression profile with increased scatter due to the expression of unique transcripts in this sample or different levels of expression of common transcripts between this sample and blastocyst 1 or blastocyst 2 (Figure 1).

### Global Gene Expression Profiles of Individual Oocytes and Blastocysts

**Figure 2:**
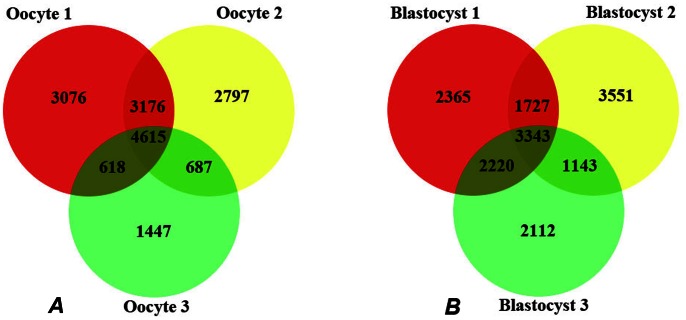
Venn diagrams showing the number of expressed transcripts unique and common to individual oocyte and blastocyst samples. A Individual oocytes expressed a number of transcripts that were unique to each one, relative to the remaining oocytes. Some transcripts were common between two individual samples and 4615 transcripts were common to 3/3 oocytes. Note that oocytes 1 and 2 shared more common transcripts with each other than with oocyte 3. These transcripts may not be exclusive to oocytes and may also be expressed in 1, 2 or all 4-cell embryo and blastocyst samples. B Individual blastocysts expressed a number of transcripts unique to each one. Some transcripts were common between two individual blastocysts and 3343 transcripts were common to all three samples. These transcripts may not be exclusive to the blastocyst stage and may also be expressed in 1, 2 or all oocyte and 4-cell embryo samples.

The scatterplot data in Figure 1 are presented as Venn diagrams in Figure 2. In the oocyte (Figure 2A), approximately 16,000 different transcripts were expressed across the three samples (i.e. in at least one), with 4615 of these (30% of the total) expressed in all 3 oocytes. Oocytes 1 and 2 each expressed over 11,000 transcripts whereas oocyte 3 expressed over 7000 transcripts. Oocytes 1 and 2 each exclusively expressed approximately 3000 transcripts, whereas oocyte 3 exclusively expressed only 1447 transcripts (Figure 2A). Oocytes 1 and 2 shared many more transcripts in common than either did with oocyte 3.

In the blastocyst (Figure 2B), again approximately 16,000 different transcripts were expressed across the three embryos, with 3343 of these (20% of the total) expressed in all three blastocysts. Individual blastocysts expressed 9655, 9764 and 8818 transcripts, respectively, with a similar degree of overlap to that seen in the oocyte samples. Blastocysts 1 and 2 shared 1727 transcripts in common (Figure 2B). Blastocyst 3 expressed 2112 unique transcripts, but shared 2220 common transcripts with blastocyst 1, more than those in common between blastocysts 1 and 2 or the 1143 shared by blastocysts 2 and 3 (Figure 2B). This analysis allows a quantitative estimate of the variability in extent of EGA in individual embryos. Blastocysts 1 and 2 express very similar numbers of genes, at 9655 and 9764 respectively (Figure 2B), but blastocyst 3 expresses considerably fewer, at 8818 genes. Assuming that the baseline level of gene expression at the 4-cell stage is very low, this means the EGA in blastocyst 3 is occurring at about 90% of the efficiency of that in blastocysts 1 and 2.

**Figure 3:**
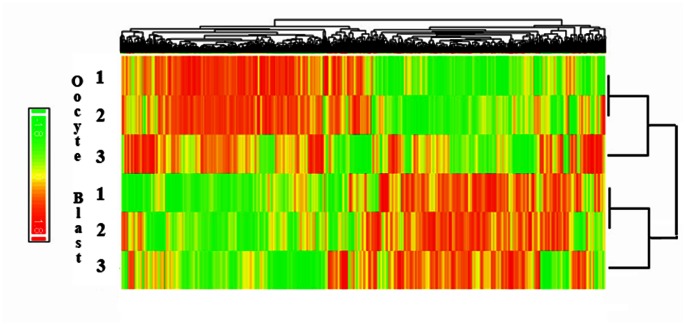
Heatmap cluster analysis of individual oocyte and blastocyst gene expression profiles. Hierarchical clustering was used to compare the gene expression profiles of individual oocytes and blastocysts, with highly expressed genes shown in red, weakly expressed in green. Oocytes show clearly distinct profiles from blastocysts, with oocytes 1 and 2 more similar to each other than to oocyte 3, and blastocyst 1 and 2 more similar to each other than to blastocyst 3. 4-cell embryos were omitted from this analysis based on the low abundance of expressed transcripts.

In order to further compare the heterogeneity in expression profiles suggested in Figures 1 and 2, and examine the similarities between same stage samples and qualitative difference between oocytes and blastocysts, we performed a heatmap cluster analysis of the three individual oocyte and blastocyst samples (Figure 3). All three oocytes clustered together and separately to the three blastocysts, as predicted. Oocytes 1 and 2 were clearly more similar to each other than to oocyte 3, and blastocysts 1 and 2 more similar to each other than to blastocyst 3, confirming the relationships observed in Figure 1.

### The Molecular Signature of Development From Oocyte To Four-cell Embryo and Blastocyst

**Figure 4:**
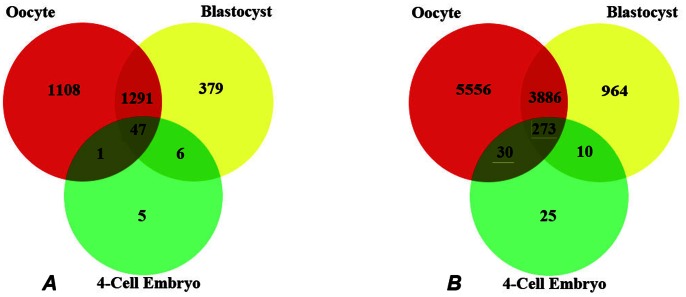
Venn diagrams showing the number of expressed transcripts unique and common to the different stages of development. **A.** A number of transcripts were uniquely expressed at a single stage: in 3/3 oocyte samples, all three 4-cell embryos and all three blastocysts. However, some transcripts were common between two different stages of development whereas some transcripts were common to all stages of development. **B** A number of transcripts were expressed in at least 2/3 oocytes, four-cell and blastocyst embryos. 273 transcripts were common to all stages of oocyte and embryo development. Over 3880 transcripts were expressed in at least 2/3 oocyte and blastocyst samples and not expressed at the four-cell stage. 30 transcripts were common to at least 2/3 oocyte and four-cell embryos and were not expressed in blastocysts. Only 4 more transcripts were shared exclusively between at least 2/3 four-cell embryos and blastocysts when compared to all four-cell and blastocyst embryos. These transcripts represent message that is transcribed early from the embryonic genome.

In order to obtain a quantitative estimate of expression of maternal transcripts, their removal by the 4-cell stage, and expression of new transcripts following EGA, we compared all three stages of development (Figure 4). We analysed genes expressed in all three individual oocytes or embryos at each stage (3/3), in order to establish “essential” baseline gene expression before and after EGA (Figure 4A). We then analysed gene expression represented in at least 2 replicates out of 3 samples (2/3), at each stage (Figure 4B). This generates a much more “permissive” dataset of gene expression, since transcripts are not excluded from the analysis because they are not called Present in one replicate sample. This also highlights the strength of an individual embryo analysis, as the difference between expression in at least 2/3 samples relative to 3/3 samples might identify the expression of genes important in conferring embryonic competence. We have not analysed genes expressed in only 1/3 samples, as it is unlikely that this would be representative of development and is not a significant advance on the previous approach of analysing data from pooled embryos.

Four-cell embryos expressed only 59 transcripts in all three embryos (Figure 4A). These were mainly ribosomal transcripts. However, the 4 cell embryo exclusively expressed Collagen and calcium binding domains EGF1 (*CCBE1*), Maternally expressed 3 *(MEG3),* the cAMP regulator, Phosphodiesterase 6B (*PDE6B)* and Natural killer tumour recognition sequence *(NKTR)*. Two other transcripts exclusive to the 4-cell embryo were identified (accession numbers AK023918 and AI133727), but have not yet been characterised. In at least 2/3 4-cell embryos, a total of 338 transcripts were expressed, with 25 transcripts coding for mainly ribosomal proteins exclusive to this stage (Figure 4B). These represent the few messages which are transcribed very early, and transiently, from the embryonic genome, and are removed from transcription by the blastocyst stage. Of the 48 transcripts shared between all oocytes and all 4-cell embryos, only 1 transcript was exclusive to both oocytes and 4-cell embryos (Figure 4A) and this transcript is currently uncharacterised (accession number AK024819). In at least 2/3 oocytes and 4-cell embryos, 303 were common but only 30 of these were shared exclusively between oocytes and 4-cell embryos (Figure 4B). These transcripts which code for genes such as RAS*-*related –GTP binding C *(RRAGC),* Sorting Nexin 24 (*SNX24*), Arginine/serine-rich_coiled-coil_2 *(RSRC2)* and Dishevelled associated activator of morphogenesis 1 *(DAAM1)* represent maternal message expressed by the oocyte but removed by the onset of EGA. Solute_carrier_family_11 member 1 *(SLC11A1),* UBA and WWE domains containing protein 1 *(HUWE1),* an alternate transcript coding for *NKTR* and three other uncharacterised transcripts (accession numbers AA639753, AU146391 and R43103) were common to all 4-cell embryos and all blastocysts (Figure 4A). Sialophorin *(SPN),* Troponin T type 3 *(TNNT3),* Syndecan 4 *(SDC4),* Adam Metallopeptidase domain 33 *(ADAM33),* Connector enhancer of kinase suppressor of RAS-2 *(CNKSR2)* and 5 other uncharacterised transcripts were common to at least 2/3 4-cell and blastocyst embryos (Figure 4B). Transcripts shared between 4-cell embryos and blastocysts but not oocytes, represent message that is newly expressed by the embryonic genome following EGA and persists to the blastocyst stage. All oocytes, 4-cell embryos and blastocysts shared 47 common transcripts (Figure 4A) whereas in at least 2/3 oocytes and embryos, 273 transcripts were common to all stages (Figure 3B). These latter transcripts represent maternal messages which persist at least to the 4-cell stage, and continue to persist to the blastocyst stage, or are re-expressed from the embryonic genome.

### Pathway Analysis of Single Human Oocytes and Blastocysts

We analysed components of 10 different pathways that were highly represented (P<0.05; q<0.22) in our individual human oocyte and blastocyst sample sets. We assessed transcripts that were significantly and uniquely represented in oocytes and blastocysts, and also those genes that were common to both oocytes and blastocysts (Table 1). In order to understand the molecular basis of oocyte and blastocyst heterogeneity and possibly developmental competence, we also assessed the differential expression of transcripts that were significantly represented in the two similar oocytes (1 and 2) relative to oocyte 3, and also in the two similar blastocysts (1 and 2) relative to blastocyst 3 (Table 2).

**Table 1:** Pathways significantly represented in all oocytes and blastocysts.

Pathway	Oocyte Specific	Blastocyst Specific	Oocyte and Blastocyst
**Apoptosis**	CIAPIN1 (NM_020313)		CYCS (BC005299)
	IKBKB (AU153366)		PRKAR2A (AK026351)
	PRKAR2A (BF246917)		
**TP53 signalling**	CD82 (NM_002231)	PERP (AJ251830)	CHEK1(NM_001274)
			CYCS (BC005299)
			RRM2 (BC001886)
**Cell Cycle**	CDC25A (AY137580)	SMC1A (BC046147)	CHEK1 (NM_001274)
	ORC5L (NM_002553)		RAD21 (NM_006265)
			TTK (NM_003318)
			CDC20 (NM_001255)
			HDAC2 (NM_001527)
			MCM6 (NM_005915)
			PCNA (NM_002592)
			YWHAB (BF246499)
			YWHAQ (NM_006826)
**Progesterone mediated oocyte maturation and oocyte meiosis**	CDC25A (AY137580)	FBXO5 (NM_012177)	CDC20 (NM_001255)
	CPEB2 (BE646645)	SMC1A (D80000)	YWHAB (BF246499)
	CALM1 (AI653730)		YWHAQ (NM_006826)
**TGFβ Signalling**	SMURF2 (AY014180)		SMAD5 (BF526175)
	SP1 (BG431266)		
	ACVR2A (AI149508)		
	ACVR2B (NM_001106)		
	FST (NM_013409)		
**Extracellular matrix and focal adhesion**	FN1 (BC005858)	ITGB3 (AI151479)	GRB2 (AF246238)
	CIAPIN1 (NM_020313)	HMMR (U29343)	ITGB1 (BG500301)
	PRKCG (AW027690)		LAMA5 (BC003355)
			LAMB1 (NM_002291)
**Purine/Pyrimidine Metabolism**	AMPD3 (NM_000480)	CTPS1 (AK025654)	APRT (AA927724)
	FHIT (NM_002012)	IMPDH1 (NM_000883)	DGUOK (BC001121)
	PDE8B (AK023913)	PFAS (AL044326)	GMPS (NM_003875)
	POLR2E (NM_002695)		POLR1B (BC004882)
	POLR3G (NM_006467)		POLR2L (BC005903)
			PRIM2 (NM_000947)
			RRM2B (AB036063)
**Valine, Leucine and Isoleucine Biosynthesis**		BCAT1 (NM_005504)	
		LARS (D84223)	
		PDHB (M34055)	
**Pyruvate metabolism**	LDHAL6A (NM_144972)	DLAT (BF978872)	PDHA1 (AW057819)
	ACYP1 (NM_001107)	PDHB (M34055)	GALM (AI769923)
**Glycolysis/Gluconeogenesis**	LDHAL6A (NM_144972)	PDHB (M34055)	LDHA (NM_005566)
		DLAT (BF978872)	PDHA1 (AW057819)

Listed entries represent transcripts and their respective NCBI accession numbers that were significantly expressed (P<0.05; q≤0.22) in the appropriate sample(s). “-“ denotes no significantly expressed components were detected in the pathway.

**Table 2:** Pathways significantly represented in oocyte and blastocyst samples with similar molecular signatures.

Pathway	Oocyte 1 & 2		Oocyte 3		Blastocyst 1 & 2		Blastocyst 3	
**Apoptosis**			BID (AA706658)		CASP9 (AA157017)			
			CASP9 (AA157017)		AKT3 (U79271)			
					MYD88 (U70451)			
					PIK3R2 (BC033311)			
					TNFRSF10A (W65310)			
**P53 signalling**	RPRM (NM_019845)		BID (AA706658)		CASP9 (AA157017)		GADD45G (NM_006705)	
			CASP9 (AA157017)		CDK2 (M68520)			
					PPM1D (NM_003620)			
					SESN2 (BF131886)			
					TNFRSF10A (W65310)			
					TNFRSF10B (AF153687)			
**Cell Cycle**	SKP2 (BG105365)		CDC23 (NM_004661)		CDK2 (M68520)		PTTG1 (NM_004219)	
	ANAPC7 (AA131793)				TFDP2 (NM_006286)		GADD45G (NM_006705)	
	ANAPC11 (BG180679)						PRKDC (U34994)	
	ANAPC13 (BC005398)							
	CDC16 (AF164598)							
	FZR1 (AA905473)							
	TFDP1 (AI950069)							
**Progesterone mediated oocyte maturation and oocyte meiosis**	CDC16 (AF164598)		CDC23 (NM_004661)		CDC25 (BC039100)		PPP2R5C (L42375)	
	ANAPC7 (AA131793)				CDK2 (M68520)			
	ANAPC11 (BG180679)				PPP1CB (NM_002709)			
	ANAPC13 (BC005398)							
	MAPK1 (AA195999)				PPP2R5E (AI689803)			
	IGF2 (M17863)							
**TGFβ Signalling**	TFDP1 (AI950069)				SMAD1 (AU146891)			
	MAPK1 (AA195999; AL157438)				SMAD6 (AI628464)			
					SMURF2 (AY014180; BE962027)			
**Pathway**		**Oocyte 1 & 2**		**Oocyte 3**		**Blastocyst 1 & 2**		**Blastocyst 3**
**Extracellular matrix and focal adhesion**		PIK3C2B (NM_002646)				SHC1 (AI809967)		COL1A2 (NM_000089)
		RAP1A (NM_002884)				COL3A1 (AU146808)		PAK1 (AU154408)
		SHC2 (AV705938)				LAMA3 (AL708055)		
		LAMA3 (AL708055)				PAK2 (BF435769)		
		MAPK1 (AA195999; AL15743)				PPP1CB (NM_002709)		
		TFDP1 (AI950069)				AKT3 (U79271)		
						PIK3R2 (BC033311; AI684344)		
**Purine/Pyrimidine Metabolism**		ADA (X02189)				ADA (X02189		DLAT (BF978872)
		ADK (U90339)				PDE6D (NM_002601)		GUCY2F (NM_001522)
		AK2 (AW277253)				POLE (AL080203)		
		AK5 (NM_012093)				POLR3C (BC004424)		
		PDE1B (NM_000924)						
		POLR2C (AJ224143)						
		POLR2G (NM_002696)						
**Valine, Leucine and Isoleucine Biosynthesis**		BCAT1 (NM_005504)		ACADSB (BE897866)		ALDH7A1 (BC002515)		
		HADHB (NM_000183)						
**Pyruvate metabolism**		LDHB (NM_002300)						
**Glycolysis/Gluconeogenesis**		ALDOA (AK026577)		PGM2 (AL136705)		ADH7 (U07821)		DLAT (AW299740)
		LDHB (NM_002300)				ALDH7A1 (BC002515)		PGM2 (AL136705)
		PGAM1 (NM_002629)						
		ALAS1 (NM00068)						
		CBS (BC007257)						
**Arginine and Proline Metabolism**						ALDH9A1 (NM000696)		
**Glycine, Serine and Threonine Metabolism**		ALAS1 (NM00068)						
		CBS (BC007257)						

Listed entries represent transcripts and their respective NCBI accession numbers common to oocyte 1 and 2 relative to oocyte 3 and blastocyst 1 and 2 relative to blastocyst 3. “−” denotes no significantly expressed components were detected in the pathway.

### Expression of Components Involved in Apoptosis, TP53 Signalling, Cell Cycle, and Progesterone Mediated Oocyte Maturation

Components of apoptosis, cell cycle and progesterone mediated oocyte maturation pathways were highly represented in the oocytes and blastocysts (P<0.05; q≤0.22) (Table 1). Pathway analysis revealed that some of these components were unique to all oocyte samples and included cytokine induced inhibitor of apoptosis 1 *(CIAPIN1),* inhibitor of kappa light chain gene enhancer in B cells, kinase of beta *(IKBKB), CD82,* cell division cycle 25A *(CDC25A)* origin recognition complex 5*(ORC5)* and cytoplasmic polyadenylation element-binding protein 2 *(CPEB2).* Relative to the oocyte, fewer transcripts were uniquely expressed in all blastocyst samples (P<0.05; q≤0.22) (Table 1). Of these genes, *SMC1A* and *FBXO5* whose expression has not previously been reported in blastocysts were significantly represented. There were a number of significantly expressed genes (P<0.05; q≤0.22) involved in these pathways that were common to all oocyte and blastocyst samples. These genes included cytochrome C somatic *(CYCS),* checkpoint 1 *(CHEK1),* ribonucleotide reductase M2 *(RRM2), RAD21, TTK,* cell division cycle 20*(CDC20),* histone deacetylase 2 *(HDAC2),* minichromosome maintenance 6 *(MCM6),* proliferating cell nuclear antigen *(PCNA),* tyrosine 3-monooxygenase/tryptophan 5-monooxygenase activation protein beta *(YWHAB)* and theta *(YWHAQ).*

Genes expressed in oocytes 1 and 2 were significantly represented (P<0.05; q≤0.22) in the cell cycle and progesterone mediated oocyte maturation and meiosis and include anaphase promoting components S-phase associated kinase 2 *(SKP2), (ANAPC)-7, -11* and *-13,* cell cycle division cycle 16 *(CDC16),* Fizzy related protein 1 *(FZR1)* and transcription factor DP1 *(TFDP1)*. Oocyte 3 uniquely and significantly (P<0.05; q≤0.22) expressed the proapoptotic factors *BID*, caspase-9 *(CASP9)* and *CDC23.*

Genes expressed in blastocysts 1 and 2 included cyclin dependant kinase 2 *(CDK2,* the MAPK-regulated component *PPM1D*, sestrin-2 (SESN2) and *TFDP2* (Table 2). The proapoptotic genes *CASP9,* tumour necrosis factor receptor superfamily member (*TNFSFR*)-*10A* and *-10B,* were also highly represented (P<0.05; q≤0.22) (Table 2). Blastocyst 3 expressed relatively fewer components that were representative of these pathways (Table 2) but genes included growth arrest and DNA-damage-inducible 45, gamma *(GADD45G),* pituitary tumour transforming gene 1 *(PTTG1)* and protein kinase DNA activated catalytic subunit *(PRKDC)*.

### Expression of Components Involved in the TGFβ Superfamily Signalling Pathway

A number of components in the TGFβ superfamily signalling cascade were significantly (P<0.05; q≤0.22) detected (Table 1). Smad specific E3 ubiquitin protein ligase 2 *(SMURF2),* the transcription factor *SP1,* activin A receptors type IIA and B *(ACVR2A, -B)* and the activin inhibitor follistatin *(FST)* were uniquely expressed in all oocyte samples. *SMAD5* was common to all oocyte and blastocyst samples (Table 1). There were no significantly (P<0.05; q≤0.22) expressed TGFβ signalling pathway transcripts that were unique to all blastocyst samples.

Oocytes 1 and 2 expressed the transcription factor *TFDP1* which has been implicated in linking TGFβ receptor activity to c-myc repression by forming a complex with smad3, E2F4/5 and p107 which translocates to the nucleus, binds to smad4 and represses c-myc expression [31]. Mitogen activated protein kinase 1 *MAPK1,* a key regulator of the MAPK signal transduction cascade as well as the TGFβ cascade is also significantly expressed by oocytes 1 and 2 (Table 2). Blastocysts 1 and 2 significantly expressed (P<0.05; q≤0.22) three members of the TGFβ signalling pathway, *SMAD1, SMAD6* and *SMURF2* (Table 2). In contrast, oocyte 3 and blastocyst 3 did not significantly express any TGFβ superfamily signalling molecules.

### Expression of Components Involved in Extracellular Matrix and Focal Adhesions

A number of cell adhesion molecules were significantly represented (P<0.05; q≤0.22) in this study. Fibronectin 1 *(FN1)* was exclusively expressed in all oocytes. *CIAPIN1,* whose expression is dependent on embryo-related growth factors such as stem cell factor (SCF) and IL-13 32 (Shibayama et al., 2004). Protein kinase C gamma (*PRKCG*), previously detected in mature human oocytes [33], was also uniquely expressed in oocytes (Table 1). Integrin β3 *(ITGB3)* is an adhesion molecules with a known role in preimplantation and peri-implantation development [34], and hyaluronan mediated mobility receptor *(HMMR),* which has been postulated to have a role in the maintenance of ESC pluripotency [35] were significantly expressed in all blastocyst samples (Table 1). Phosphatidylinositol 3-kinase regulatory subunit 2 (*PIK3R2*), integrin β1 *(ITGB1),* laminin α5 *(LAMA5)* and Laminin β1 *(LAMB1)* involved in cell –extracellular matrix interaction and signalling were significantly represented (P<0.05; q≤0.22) and common to all oocyte and blastocyst samples (Table 1).

Several transcripts involved in the extracellular and focal adhesion signalling cascades were significantly expressed in oocytes 1 and 2 relative to their third counterpart. Phosphatidylinositol 3-kinase class 2 β *(PIK3C2B),* which has been previously shown to regulate cell mobility by reorganising the actin cytoskeleton [36], RAS-related protein 1A *(RAP1A),* was detected in human oocytes [37] and is postulated to play a role in regulating normal morphogenesis [38], SHC transforming protein 2 *(SHC2),* laminin α3 *(LAMA3)* and the previously mentioned *MAPK1* and *TFDP1* were highly represented in oocytes 1 and 2 (Table 2). Oocyte 3 did not significantly express transcripts that were highly represented in these pathways (Table 2). Blastocysts 1 and 2 also expressed *LAMA3* together with *SHC1,* collagen type III α1 *(COL3A1),* P21*-*activated kinase 2 *(PAK2),* protein phosphatase 1β *(PPP1CB), AKT3* and Phosphatidylinositol 3-kinase regulatory subunit 2 *(PIK3R2)* (Table 2). Only two transcripts pertaining to extracellular matrix-adhesion signalling cascades, *COL1A2* and *PAK1*, were significantly represented (P<0.05; q≤0.22) in blastocyst 3 (Table 2).

### Expression of Components Involved in Purine and Pyrimidine, Amino Acid and Carbohydrate Metabolism

Purine and pyrimidine metabolism are important for the synthesis of new ribonucleotides during the process of meiosis, cell division, protein synthesis and DNA repair. Components of these pathways were significantly expressed (P<0.05; q≤0.22) in all oocyte and blastocyst samples. Adenosine monophosphate deaminase 2 *(AMPD3),* Fragile histidine triad gene *(FHIT),* phosphodiesterase 8B polymerase II RNA subunit E *(POLR2E)* and polymerase II RNA subunit G *(POLR3G)* were amongst those transcripts uniquely expressed in all oocytes. CTP synthetase *(CTPS1),* inosine-5-prime-monophosphate dehydrogenase1 *(IMPDH1)* and phosphoribosylformyl-glycinamidine synthase *(PFAS)* were uniquely expressed in all blastocyst samples. Some components of these pathways were common to all oocyte and blastocyst samples and include adenosine phosphoribosyltransferase *(APRT),* deoxyguanosine kinase *(DGUOK)* guanine monophosphate synthetase *(GMPS),* polymerase 1 RNA subunit B *(POLR1B),* polymerase II RNA subunit L *(POLR2L),*primase polypeptide 2A *(PRIM2)* and ribonucleotide reductase M2 B *(RRM2B)* (Table 1). Oocytes 1 and 2 expressed a number of components that were highly represented in the ribonucleotide synthesis pathways (P<0.05; q≤0.22) including adenosine deaminase *(ADA),* adenosine kinase *(ADK),* adenylate kinase-2 and 5 *(AK2, -5)* and phosphodiesterase 1B *(PDE1B).* In contrast, oocyte 3 did not significantly express unique components of this pathway (Table 2), suggesting that it might have reduced developmental competence. Blastocysts 1 and 2 also significantly expressed adenosine deaminase (*ADA)*, together with *PDE6D,* polymerase DNA epsilon *(POLE)* and polymerase (RNA) III (DNA directed) polypeptide C *(POLR3C).*

Components of the pyruvate metabolism pathway were significantly expressed in oocytes and blastocysts (Table 1). Lactate dehydrogenase A-like 6A *(LDHAL6A),* acylphosphatase erythrocyte *(ACYP1)* were unique to all oocyte samples whereas the previously mentioned *DLAT* together with pyruvate dehydrogenase, beta polypeptide B *(PDHB)* were significantly (P<0.05; q≤0.22) expressed and found only in all blastocyst samples. Pyruvate dehydrogenase complex, E1 alpha polypeptide 1 *(PDHA1)* and galactose mutarotase *(GALM)* expression was common to all oocyte and blastocyst samples (Table 1). In contrast, when comparing stage-specific differential expression of pyruvate metabolic components within individual samples, oocytes 1 and 2 were the only samples to express one transcript representative of the pyruvate metabolism pathway, lactate dehydrogenase B *(LDHB)* (Table 2).

Our study also demonstrated significant expression (P<0.05; q≤0.22) of a number of components involved in valine, leucine and isoleucine metabolism. Interestingly, few transcripts were expressed in all 3/3 oocytes (Table 1). In contrast, a number of transcripts were expressed in all 3/3 blastocyst samples (Table 1). Only one transcript, *PDHA1* was significant and common to all oocyte and blastocyst samples (Table 1). Branched chain aminotransferase 1 *(BCAT1)* and hydroxyacyl-CoA dehydrogenase *(HADHB)* were unique to oocytes 1 and 2, relative to the third oocyte sample (Table 2). Oocyte 3 uniquely expressed acyl-CoA dehydrogenase short/branched chain *(ACADSB)* and blastocysts 1 and 2 expressed alcohol dehydrogenase 7 A1 *(ALDH7A1)* (Table 2).

Components of arginine, proline, glycine, serine and threonine metabolic pathways were not detected at significant levels in all oocyte and blastocyst samples. However, oocytes 1 and 2 did significantly express two pathway components, delta-aminolevulinate synthase 1 *(ALAS1)* and cystathionine beta-synthase *(CBS)* and *ALDH9A1* was significantly expressed in blastocysts 1 and 2.

Components of the glycolytic pathway were found to be significantly expressed in oocytes and blastocysts (Tables 1 and 2). *LDHAL6A* was unique to all oocyte samples whereas *PDHB* and *DLAT* were unique to all blastocyst samples. *LDHA* and *PDHA1* were significantly expressed in all oocyte and blastocyst samples (Table 1).

Oocytes 1 and 2, but not 3 expressed a number of significantly represented glycolytic pathway components (P<0.05; q≤0.22) (Table 2), including aldolase A fructose-bisphosphate *(ALDOA), LDHB,* phosphoglycerate mutase 1 *(PGAM1)* and *CBS*. Oocyte 3 expressed one unique transcript at a significant level (P<0.05; q≤0.22), phosphoglucomutase 2 *(PGM2).* Blastocysts 1 and 2 uniquely expressed alcohol dehydrogenase 7 *(ADH7)* and *ALDH7A1* while *DLAT* and *PGM2* were uniquely expressed by blastocyst 3 (Table 2).

## Discussion

We describe a global analysis of gene expression at stages spanning human preimplantation development, at the level of the individual oocytes/embryos. The variability in gene expression which we have found between oocytes and embryos at the same stage requires the re-interpretation of previous microarray studies based on pooling a number of oocytes and embryos at each developmental stage. This practice, common in studies of animal embryos where development is relatively homogeneous, has unfortunately obscured the heterogeneity in development which is a hallmark of early human embryos. Understanding this is key to understanding the molecular basis of early human development, the establishment of developmental competence and for distinguishing the molecular fingerprints of viable and non viable embryos in assisted reproduction treatments. Previous data from pooled embryos represent averages of individual sample transcripts and are likely to be highly unrepresentative of normal development. False negative results arise since high expression of an important gene may be an important marker of viability, but be diluted out by lack of expression in non viable embryos, or conversely, false positives will arise when only one individual embryo sample of a pool provides the transcript contribution and apparent expression of a gene in the pool.

Our approach has identified a number of molecular pathways that are exclusive to each developmental stage or alternatively common amongst different stages, and revealed differences in gene expression between individual human oocytes and blastocysts. Our approach furthermore provides a quantitative estimate of the extent of embryonic genome activation, by comparing transcription between developmental stages, and the extent to which this varies between individual embryos. We identified components that were unique to each individual sample, and we propose that some transcripts may represent potential markers of oocyte and embryo competence and viability. Of course expression of mRNA transcripts does not necessarily imply translation of that gene product to a protein product, nor does it provide information on post translational processing or function such as enzyme activity. These require detailed follow-up studies using assays with sensitivity sufficient for single embryos, e.g. protein localisation studies [23], enzyme activity studies [39], or systems biology approaches such as metabolomics [40], [41].

We identified a number of stage-specific transcripts, transcripts detected in common between developmental stages as well as those unique to particular samples. Oocytes, four-cell embryos and blastocysts were all enriched in transcripts for ribosomal pathways and protein synthesis. These transcripts represent maternal message which may persist to the blastocyst stage, or which may be degraded and re-expressed after EGA [1], [3], [42]. Only one (uncharacterised) transcript which was not expressed in blastocysts was common to all oocytes and four-cell embryos. In at least 2/3 oocytes and four-cell embryos, 30 transcripts were shared but not expressed by blastocysts representing maternal message that were not re-expressed by the embryonic genome. There were 6 transcripts shared by all four-cell and blastocyst embryos (but not by oocytes) and 10 that were Present in at least 2/3 four-cell and blastocysts. These transcripts represent mRNAs that are expressed from 4-cell EGA though the preimplantation period and include *CCBE1*, a tumour suppressor gene with a suggested role in extracellular matrix remodelling and the cyclic AMP antagonist *PDE6B* responsible for removing cAMP. One transcript coding for the maternal imprinted gene *MEG3* (accession number AI133721) was also exclusively expressed in all four-cell embryos. *CCBE1, PDE6B* or *MEG3* have not previously been reported in human preimplantation embryo development. Croteau et al [43] reported one isoform of *MEG3* expression in mouse oocytes and two-cell embryos. In our data other transcripts coding for *MEG3* were also detected in 2/3 oocytes (accession number AI950273) and 1/3 blastocysts (accession number BF956762). The variable expression of this gene emphasises the importance of using single oocytes and embryos for analysis.

### Cell Cycle Regulation and an Oocyte Signature

The importance of several components involved in apoptosis, cell cycle and progesterone mediated oocyte maturation pathways in the development of competent oocytes and embryos have been reported in previous studies [22], [42], [44]–[50] and Assou et al [51] used microarray to assess gene expression in pooled human oocytes and human embryonic stem cells (hESCs) as a model for early embryonic development. Assou et al [51] described a unique oocyte signature comprising of *DAZL, SOX30, AURKC* and *PTTG3P*, amongst other transcripts and these were significantly expressed in oocytes in our study. Components of this signature comprised *CHEK1, FBXO5, CDK7* and *CDK8*. Our data showed that *CHEK1,* was significantly expressed in all oocytes and blastocysts. This kinase is postulated to inhibit CDC25C in the event of DNA damage, thus preventing activation of the CDC2-cyclin B complex and entry to mitosis [52]. Zhang et al [42] assessed the gene expression profiles of human germinal vesicle oocytes relative to hESCs and foreskin fibroblasts and identified *GDF9* and *ZP2* and *MOS* as oocyte-specific genes highly represented in hGVOs. These genes were also expressed at a significant level in oocytes in our study; however, *MOS* was only expressed in oocyte 3. *BMP6, ZP1, ZP4 POMZP3, ZAR1, NLRP5* and *FIGLA* were highly represented in oocytes and down-regulated at the blastocyst stage. With the exception of *BMP6* (all blastocysts only) and *ZP1* (1/3 oocytes and all blastocysts), our study is consistent with these findings. However our data identify differences in expression compared to the above studies, again emphasising the importance of analysing single embryos to avoid the misleading averaging effect of pooled samples.

### TGFβ Superfamily

The TGFβ superfamily signalling pathway has been implicated in many biological and developmental processes including folliculogenesis, oocyte maturation and early embryogenesis [42], [53]–[56] and differentiation of embryonic stem cells [57]. Our study found that *SMURF2, SP1, ACVR1, ACVR2* and *FST* were enriched in all oocyte samples and *SMAD5* was enriched in all blastocysts. *ACVR1* and *FST* expression have been detected in the cumulus cells of cumulus-oocyte-complexes (COCs) from both *in vitro* and *in vivo* matured oocytes [58]. Vandevoort et al [58] and Lee et al [53] have shown a direct link between the levels of FST and oocyte competence in terms of increased blastocyst formation rate, increased total blastocyst cell number and increased total trophoblast cell number. Components of the TGFβ signalling cascade were enriched in oocytes 1 and 2 and blastocysts 1 and 2. In light of the known importance the TGFβ cascade in oocyte competence and early embryogenesis (reviewed in [54]) the expression of these molecules in these samples relative to oocyte 3 and blastocyst 3 may be an important indicator of their developmental competence.

### Adhesion Receptors and the Extracellular Matrix (ECM)

Extracellular matrix molecules are important in the formation of a fully differentiated, implantation competent blastocyst [59], [60]. Adhesion proteins also have important roles in the maintenance of pluripotency and ES cell differentiation [61] D. Soteriou, D. Brison, SJ Kimber in prep). In common with previous studies, we have identified a number of constitutively expressed and stage specific adhesion receptors and ECM molecules. Integrin *β1* (*ITAB1)* was expressed in all oocytes and all blastocysts whereas *ITAB3* was exclusive to all blastocysts. However, transcripts for the binding partner to ITAB3, αv integrin (ITAV) were only detected in blastocyst 2 and oocytes 2 and 3. Since *ITAB3* is detected only post 4-cell, it is likely the ITAV protein in oocytes binds to an alternative partner, possibly ITAB5, which was expressed in all oocytes and blastocysts. Integrin αvβ5 binds to fibronectin (as well as other apparently non expressed molecules e.g. vitronectin), and we found *FN1* expressed at significant levels in all oocytes and one transcript representative of *FN1* (accession number W73431) was detected in blastocysts 2 and 3 but this was not significant. Integrin αvβ3 binds to laminin and *LAMB1* and *LAMB5* were common to all oocytes and blastocysts. It has been postulated that ECM molecules may act as bridging proteins to bring the TE to the luminal surface of the uterus for implantation [23], [62]. However, FN1 and LAMB1 null murine embryos still implant, although LAMB1 null embryos die after implantation due to failure of endoderm differentiation [63], [64].

### Metabolic Pathways

Previous reports have investigated the metabolic profile of oocytes and preimplantation embryos as a non-invasive method to identify competent oocytes and viable embryos with a view to utilising this in assisted reproductive technologies [65]–[71].

Pyruvate, synthesised via the metabolism of glucose, and glucose itself, are the major sources of energy for preimplantation development and mature oocytes [72 73]. The culture of oocytes in sub-optimum glucose conditions have been suggested to result in failure of resumption and completion of meiosis, a decrease in cytoplasmic maturation and reduced developmental potential [74]–[76]. Moreover, glycolysis results in the production of pyruvate and phosphoribosylpyrophosphate, the latter forms the substrate for *de novo* purine synthesis [73]. Our study found components of the glucose metabolism pathway in all oocytes and blastocysts. However, components were also significantly represented in each sample. Oocytes 1 and 2 expressed a different cohort of glucose metabolism components than oocyte 3. A similar pattern was observed for blastocysts 1 and 2 relative to 3. Although different transcripts of the glucose metabolism pathway were enriched in each sample, it is unknown whether expression of different genes was indicative of oocyte or blastocyst viability.

Amino acid turnover by preimplantation embryos is related to embryo developmental competence and clinical outcome in ART [68 69]. Houghton et al [68] related amino acid profile to blastocyst formation and showed that during embryo culture, Leu was continuously depleted from the media and Glu and Ala was synthesised by embryos on day 2/3 that went on to reach the blastocyst stage. After day 3, Leu depletion was accompanied by Arg, Ser, Met and Val depletion in embryos that reached blastocyst stage. Brison et al reported a significant depletion of Leu and Ser from the media indicative of embryos that will give rise to pregnancy. In contrast to Houghton et al, this profile was accompanied by a decrease in Gly and increases in Asn and Arg [68], [69]. We found components of the Leu metabolism pathway significantly enriched in oocytes and blastocysts. Components of Ser metabolism were also expressed in oocytes 1 and 2 suggesting again that these are competent relative to oocyte 3. Interestingly, no components in the Ser metabolic pathway were significantly expressed in blastocysts, suggesting that this viability is conferred by inheritance of maternal message.

## Materials and Methods

### Embryos

Human oocytes and embryos were donated to research after fully informed patient consent in writing, with approval from Central Manchester Research Ethics Committee and the Human Fertility and Embryology Authority (research licence R0026). Fresh oocytes and embryos surplus to IVF requirement were obtained from Saint Mary’s Hospital, Manchester. Basal characteristics of the patients donating embryos are as detailed in Roberts et al. [77]. For ethical reasons it was only possible to obtain failed to fertilise oocytes for analysis. Failed to fertilise oocytes were obtained 24h after insemination and after removing any contaminating cumulus cells the oocytes were lysed immediately for polyAPCR amplification as previously described [26], [27]. All embryos were surplus to infertility treatment and developmentally scored, according to standard clinical grading systems used at St Mary’s Hospital [78]. All embryos scored ≥3 for equal blastomere size and ≥3 for level of fragmentation and their speed of development was normal. Early cleavage embryos were obtained at the two-four cell stage on day 2 of development and cultured to the four- and eight-cell stage in 50 µl drops of G1 medium (Vitrolife, UK) overlaid by liquid paraffin (Medicult UK Ltd, UK). Embryos at the eight-cell stage were transferred to 50 µl drops of G2 medium (Vitrolife, UK) overlaid with liquid paraffin and cultured from the eight-cell stage to the blastocyst stage. Blastocysts were graded using the Gardner and Schoolcraft method [79] and only blastocysts with the minimum grade of 5BB were used.

### Embryo Lysis, Reverse Transcription, Global Amplification (PolyAPCR) and Hybridisation to Human Genome U133 Plus 2.0 Arrays

Oocytes and embryos were lysed and reverse transcribed as previously described [23], [27]. PolyAPCR was performed to amplify mRNA, as described by Brady and Iscove [26]. This procedure amplifies all polyadenylated RNA in a given sample. The cDNA collection thus produced preserves the relative abundance of the mRNAs present in the original sample [80]–[82]. PolyAcDNA was then subjected to a second round of amplification and biotin-16-dUTP labelling using EpiStem’s proprietary PolyAPCR based systems, EpiAmp™ (PolyAPCR based amplification) and EpiLabel™ (PolyA-PCR labelling), according to the Manufacturer’s instructions. All samples were assayed for expression of β-actin as the expression of this gene was our minimum inclusion criteria for microarray analysis. Labelled PolyAcRNA was hybridised to Affymetrix Human Genome U133 Plus 2.0 arrays.

### Analysis and Normalisation of Gene Expression Data

Gene expression analysis and normalisation MAS 5.0 method (Affymetrix. Affymetrix Microarray Suite User Guide. Affymetrix, Santa Clara, CA, version 5 edition, 2001) were performed using Bioconductor [83]. Differential expression analysis was performed using Limma using the functions lmFit and eBayes [84]. Genelists of differentially expressed genes were controlled for false discovery rate (fdr) errors using the method of QVALUE [85]. Hierarchical clustering was performed on a subset of 10,432 probesets that were Present in at least 2/3 samples of oocyte or blastocyst using Partek Genomics Solution (version 6.3, Copyright 2005, Partek Inc., St. Charles, MO, USA). We deliberately set the threshold for calling a gene present on the array conservatively, in order to minimise the rate of false negative data. As a result of this of course, low level expression of some genes may be not called present. Clustering was performed on gene expression values of each sample group (log 2) that had been z-transformed (for each probeset the mean set to zero, standard deviation to 1).” All microarray data is MIAME compliant and has been deposited with EMBL-EBI (accessed at http://www.ebi.ac.uk; accession number E-MEXP-3870).

Analysis and interpretation of the data was performed using the functional annotation tool of the Database for Annotation, Visualization and Integrated Discovery (DAVID) 2.1 programme [86].

### Summary

Analysis of individual oocytes, four-cell embryos and blastocysts has given us insight into the molecular signature underpinning human preimplantation development. Our study has highlighted the importance of using single oocytes and embryos in order to understand the heterogeneity inherent in human development, and to identify potential markers or pathways indicative of competence and viability. The substantial differences in transcript number in each categories of the Venn diagrams (Figure 4) between the 3/3 data and the 2/3 data illustrates the magnitude of the variation between morphologically similar oocytes/embryos at each stage. Previously published microarray data using pooled samples of oocytes/embryos may have skewed the data and masked these differences. Although a number of pathways were represented in our microarray readout, we found more components representative of cell cycle regulation, adhesion receptor/ECM and regulation of purine, pyrimidine and amino acid metabolism in oocytes 1 and 2 than oocyte 3. These results may suggest that oocyte 3 had reduced developmental competence. However our previous research on the chemical activation of failed to fertilise oocytes demonstrates some of these oocytes can generate blastocysts and embryonic stem cell lines [29], [30], [87]. Similarly, blastocysts 1 and 2 expressed more components of these pathways than blastocyst 3 again suggesting some compromise in the latter, especially as some of these components are required for TE differentiation and extracellular matrix modelling, which are required for successful implantation and amino acid metabolism, an important marker of embryo viability. Our data provides additional information on the temporal and differential gene expression profile of individual human oocytes and preimplantation embryos and can be utilised as a basis for further investigation to aid identification of viable embryos for transfer in ART, the health of ART children and understanding the basis of pluripotency in stem cell lines.
